# The Effect of Micronutrients on Obese Phenotype of Adult Mice Is Dependent on the Experimental Environment

**DOI:** 10.3390/nu16050696

**Published:** 2024-02-29

**Authors:** Zeyu Yang, Ruslan Kubant, Eva Kranenburg, Clara E. Cho, G. Harvey Anderson

**Affiliations:** 1 Department of Nutritional Sciences, Temerty Faculty of Medicine, University of Toronto, Toronto, ON M5S 1A8, Canada; zey.yang@mail.utoronto.ca (Z.Y.); ruslan.kubant@utoronto.ca (R.K.); eva.kranenburg@mail.utoronto.ca (E.K.); 2 Department of Human Health and Nutritional Sciences, College of Biological Science, University of Guelph, Guelph, ON N1G 2W1, Canada; claracho@uoguelph.ca; 3 Department of Physiology, Temerty Faculty of Medicine, University of Toronto, Toronto, ON M5S 1A8, Canada

**Keywords:** micronutrients, diet, obesity, insulin resistance, gene expression, experimental environment

## Abstract

The environment of the test laboratory affects the reproducibility of treatment effects on physiological phenotypes of rodents and may be attributed to the plasticity of the epigenome due to nutrient-gene-environment interactions. Here, we explored the reproducibility of adding a multi-vitamin-mineral (MVM) mix to a nutrient-balanced high-fat (HF) diet on obesity, insulin resistance (IR), and gene expression in the tissues of adult male mice. Experiments of the same design were conducted in three independent animal facilities. Adult C57BL/6J male mice were fed an HF diet for 6 weeks (diet induced-obesity model) and then continued for 9–12 weeks on the HF diet with or without 5-fold additions of vitamins A, B1, B6, B12, Zn, and 2-fold Se. The addition of the MVM affected body weight, fat mass, gene expression, and markers of IR in all three locations (*p* < 0.05). However, the direction of the main effects was influenced by the interaction with the experimental location and its associated environmental conditions known to affect the epigenome. In conclusion, MVM supplementation influenced phenotypes and expression of genes related to adipose function in obese adult male mice, but the experimental location and its associated conditions were significant interacting factors. Preclinical studies investigating the relationship between diet and metabolic outcomes should acknowledge the plasticity of the epigenome and implement measures to reproduce studies in different locations.

## 1. Introduction

The results of more than 50 percent of preclinical research cannot be reproduced [1,2]. In recent years, many publications have identified the environment in which the studies are conducted as a significant and overlooked factor [3,4,5,6]. While attempts to mitigate differences in the environment are recommended, it is also acknowledged that many factors cannot be controlled. Expert committees have noted that introducing heterogenization rather than standardization of laboratory animal research can improve reproducibility as well as add confidence to responses to experimental treatments [7,8]. Thus, to provide confidence in treatment outcomes, studies should be repeated in an environment separate from the primary study. A plausible explanation offered for the effects of the environment rests with the epigenome [4], which is much more plastic than the genome itself and results in modifications of gene expression and function [9].

The epigenome consists of chemical modifications to DNA or chromatin structures that alter gene expression without changing the DNA sequence itself [9]. Until recently, it was thought that the epigenome is established during early development and then remains tightly regulated throughout the life span; however, recent reports have revealed that environmental factors, including diet, can change epigenetic patterns that program the phenotype post-birth [10]. Environmental factors that have been identified to affect the growth, behaviors, and neural function of the experimental animals include the animal facility housing conditions [11], diet [12], sex of the personnel [13], size and color of the cage [14], and their neighboring rearing cages [5]. These factors are likely to be modifiers of the regulatory function via several epigenetic mechanisms, including methylation that alters gene expression [15].

In our recently published study, we added a multivitamin-mineral (MVM) mix containing 5-fold amounts of vitamin A, B1, B6, B12, and zinc (Zn), and 2-fold selenium (Se) to a high-fat diet fed to adult male mice [16]. The novel and surprising observation was that the MVM reduced weight gain insulin resistance (IR) and modified the expression of genes associated with IR in white adipose tissue and liver. It also shifted one-carbon metabolism in the liver toward favoring increased gene methylation [16]. Because of the recently expressed concern with the reproducibility of scientific research, we conducted two more experiments of a similar design but at different animal facilities to test the replicability of the MVM’s effect on characteristics of metabolic syndrome.

We hypothesized that the physiological benefits of the MVM added to the high-fat diet of mice with diet-induced obesity (DIO) would be reproduced in all animal facilities. The objective of this report was to show the effect of the experimental environment in animal facilities on the reproducibility of the physiological outcomes of adding MVM to a high-fat diet in mice with DIO.

## 2. Materials and Methods

### 2.1. Experimental Design

Ethical approval for all experimental procedures was obtained from the University of Toronto Office of Research Ethics (protocol no. 20012670 and no. 20012824) and the Animal Care Committee of The Centre for Phenogenomics (protocol no. 25-0372H).

The experiments of the same design were conducted between December 2020 and October 2022 in three independent research facilities. Experiment 1 was conducted in the Department of Comparative Medicine (DCM) at the University of Toronto. Experiment 2 was conducted at The Centre for Phenogenomics (TCP) in the Mount Sinai Hospital. Experiment 3 was conducted at the Terrence Donnelly Centre for Cellular and Biomolecular Research (CCBR) at the University of Toronto.

For each experiment, 10-week-old male mice (inbred C57BL/6J DIO, Stock No: 380050) were purchased from Jackson Laboratory (Bar Harbor, ME, USA) and delivered to the respective animal facilities (locations) in December of 2020 (DCM), February (TCP) and June (CCBR) of 2022. Upon arrival, mice were housed in accordance with facility regulations, with four mice per cage at DCM and CCBR and five mice per cage at TCP. Housing conditions included a 12:12-h light-dark cycle (lights on at 0700) with a temperature maintained at 22 ± 1 °C. Mice had ad libitum access to food and water during a two-week acclimatization period in each experiment.

Mice were weighed upon arrival and subsequently on a weekly basis throughout the duration of each experiment. Body weight (BW) gain was calculated as the difference from the initial body weight. At the end of each experiment, following a 6-h daytime water-only fasting period, mice were anesthetized with isoflurane inhalation and subjected to cardiac puncture for blood collection. Euthanasia was administered using cervical dislocation, and tissues were collected. Blood samples were centrifuged at 4 °C for plasma separation. Epididymal white adipose tissue (eWAT) and liver were dissected, weighed, and preserved. Both plasma and tissues were snap-frozen and stored at −80 °C for future analyses. eWAT fat pad mass and liver weight (g) were adjusted relative to body weight at termination (g) and expressed as a percentage.

### 2.2. Dietary Interventions

After a two-week acclimatization period, 12-week-old male mice were randomly assigned via random number generators (GraphPad Software, version 9.5.1, Inc., Boston, MA, USA) to one of the two diets: (1) a high-fat (60 kcal%) diet containing the recommended AIN-93M vitamin and mineral content served as the control (i.e., the HF group), or (2) an HF diet supplemented with 5-fold the recommended for the AIN-93M diet amounts of vitamins A, B1, B6, B12, Zn, and 2-fold Se, which was the treatment diet (i.e., the HF-MVM group). Mice were maintained on their corresponding diets for either 9 weeks (DCM, *n* = 12/group, 24 mice in total), 10 weeks (TCP, *n* = 15/group, 30 mice in total), or 12 weeks (CCBR, *n* = 12/group, 24 mice in total). Cages were alternatively placed between HF and HF-MVM groups on the rack to minimize confounders. Detailed formulations of the HF and HF-MVM diets used in all three experiments are provided in Supplemental Table S1. All six experimental diets were customized and purchased from Research Diets (New Brunswick, NJ, USA). In the TCP, both HF and HF-MVM diets underwent radiation treatment as part of the facility’s mandatory pathogen contamination control procedures. The main experimenter could not be blinded to the experimental groups due to the difference in the colors of the diets, but other independent investigators were involved in procedures, such as body weight measuring, insulin tolerance test, tissue sampling, data collecting, and analyzing to minimize subjective biases.

To confirm the micronutrient content of MVM as well as the stability of dietary lipid throughout the experiments, the targeted micronutrient content and lipid peroxidation levels of both HF and HF-MVM diets were analyzed by Eurofins Experchem Laboratories Inc. (North York, ON, Canada) after the experiments. Vitamin A, B1, B6, B12, and Zn contents in the MVM were found to be highly stable and consistent across the three experiments (Supplemental Table S2). Free fatty acid (FFA) content was analyzed using gas chromatography (GC) methods (Supplemental Table S3).

### 2.3. Insulin Tolerance Test (ITT) and Insulin Resistance (by-HOMA-IR)

After 7–8 weeks on the experimental diets, mice underwent an intraperitoneal insulin challenge (1.5–2.0 IU of insulin per kg BW) following a 6-h water-only fast. Prior to insulin injection, baseline fasting glucose levels were measured from venous blood samples obtained via saphenous tail veins using an Accu-Chek^®^ Aviva glucometer (Roche Diagnostics, Laval, QC, Canada). Approximately 100 µL of blood was collected into heparin-coated capillary tubes (Cat# 20.1309.100, Sarstedt, Nümbrecht, Germany) for plasma analysis. Post-injection glucose levels were assessed at specific time points: 10, 20, 60, 90, and 120 min at TCP and 15, 30, 60, 90, and 120 min at DCM and CCBR. The rate constant for glucose disappearance (kITT) was calculated over the initial 15 min (DCM and CCBR) and 20 min (TCP) using the formula [17]:

kITT (%min/1) = (0.693/t1/2) × 100

Plasma samples were analyzed using commercially available ELISA kits to determine fasting insulin concentrations (Cat# 80-INSMSH-E01, Alpco Diagnostics, Salem, NH, USA). The homeostatic model assessment for insulin resistance (HOMA-IR), a proxy marker of insulin resistance based on fasting glucose and insulin levels, was calculated using the following equation [17]:

HOMA-IR = fasting glucose (in mmol/L) × fasting insulin (in μU/mL)]/22.5

### 2.4. RNA Isolation and Gene Expression

The effect of addition of MVM to the HF diet on the expression of the same genes related to adipose functions, including those involved in lipogenesis pathway (sterol regulatory element binding transcription factor 1 (*Srebf1*), fatty acid synthase (*Fasn*), acetyl-CoA carboxylase alpha (*Acaca*), and stearoyl-CoA desaturase 1 (*Scd1*)), adipogenesis (peroxisome proliferator activated receptor gamma, (*Pparg*)), and synthesis of certain adipokine (adiponectin (*Adipoq*), leptin (*Lep*), and retinol binding protein 4 (*Rbp4*)) were measured in eWAT and upstream insulin signaling genes (phosphoinositide-3-kinase regulatory subunit 1 (*Pik3r1*) and thymoma viral proto-oncogene 1 (*Akt1*)) in the liver in the three experiments.

Chloroform (Sigma-Aldrich, Darmstadt, Germany) extraction was used to isolate RNA from homogenized samples (150 mg for eWAT or 50 mg for liver) in Trizol reagent (Invitrogen, Grand Island, NY, USA). The extracted RNA samples, quantified at 500 to 1000 ng, were of good quality with the 260/230 nm ratio of 2.0–2.2 and 260/280 nm ratio of 2.0 using the NanoDropTM2000 Spectrophotometer (Thermo Fisher Scientific, Inc., Waltham, MA, USA), and was utilized for cDNA synthesis. The synthesis process was conducted using the High-Capacity cDNA Archive Kit (Applied Biosystems Inc.; ABI, Foster City, CA, USA) on the TProfessional Standard Gradient 96 thermocycler (Biometra, Analytik Jena, Jena, Germany). Subsequently, equal volumes of cDNA (1 μL/sample) were utilized to quantify gene expression levels via real-time PCR using a QuantStudio System (Thermo Fisher Scientific, Inc., Waltham, MA, USA) and TaqMan Gene Expression Master Mix and Assays (Cat# 4369016 Thermo Fisher Scientific, Inc., Waltham, MA, USA). The primers (TaqMan Assays) were ordered from Thermo Fisher Scientific, and all the inventory information is listed in Supplemental Table S4. Gene expression results were analyzed using 2^−ΔΔCT^ (cycle threshold) method [18], with expression levels for each gene being normalized to the housekeeping gene *Tbp* (TATA-box-binding protein).

### 2.5. Statistical Analysis

Data analyses were performed using SAS Version 9.4 software (SAS Institute Inc., Carey, NC, USA) and R Studio (Version 1.4.1106, Posit Software, Boston, MA, USA). The variance across groups was homogeneous and normally distributed, tested using Levene’s test. A sample size of 5 mice per group has been deemed sufficient to detect a 10% difference in gene expression, our primary outcome measure, at a significance level of *p* < 0.05 and a power of 0.80 (by G Power software, version 3.1) based on our previous data [16,19]. However, a sample size of 12 per group was needed to detect 10% differences among treatment groups for other metabolic and phenotypic measures, such as BW. Outliers were identified and excluded using robust regression analysis. To assess the effects of BW gain over time and glucose response during the ITT, a two-way analysis of variance (ANOVA) was conducted using the PROC MIXED procedure, with MVM (non-MVM vs. MVM) and time as primary factors, as well as an MVM*time interaction term. Subsequently, Tukey’s post hoc tests were applied. Student *t*-tests were employed to compare final BW, insulin, and tissue measurements and relative mRNA expression between the HF and HF-MVM groups within each experiment. To test the effect of the test environment (e.g., study location) as a potential confounder that might interact with the MVM, a two-way ANOVA was conducted with MVM (with or without) and Location (DCM, TCP, or CCBR) as the main factors, accompanied by an MVM*Location interaction term. Significant interactions were subjected to Tukey’s post hoc analysis, adjusted for multiple comparisons. All values are presented as mean ± standard error of the mean (SEM). Statistical significance was set at *p* ≤ 0.05.

Data presented from the DCM study utilize figures or reformatted data derived and adapted from our previous publication [16].

## 3. Results

### 3.1. Body Weight Gain

**Figure 1:**
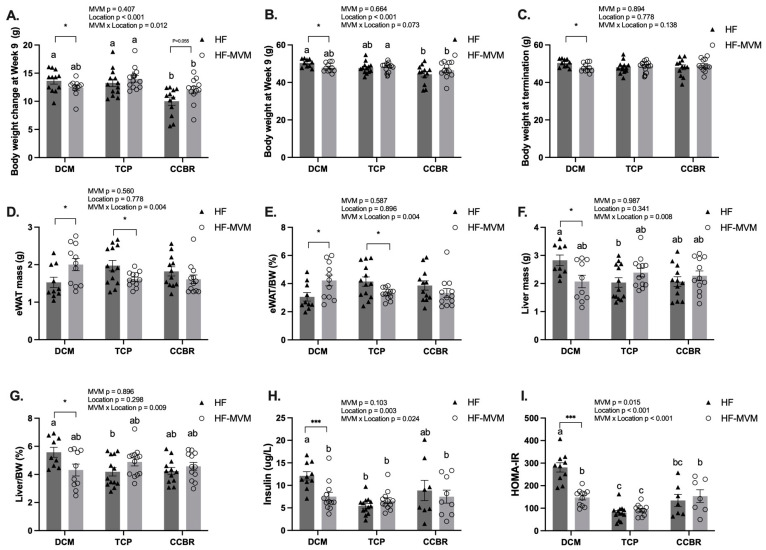
Phenotypical measures in three experiments. Body weight change at Week 9 (**A**), body weight at Week 9 (**B**) and at termination in each study (**C**), eWAT mass (**D**) and percentage to body weight (**E**), liver mass (**F**) and percentage to body weight (**G**), fasting insulin levels (**H**) and HOMA-IR (**I**) across the three studies. Values are Mean ± SEM, *n* = 9–12 for DCM and CCBR, and 12–15 in TCP. A two-way ANOVA was conducted with Location (DCM, TCP, and CCBR) and MVM (HF or HF-MVM) as main factors and an MVM × Location interaction term. A Tukey’s post hoc analysis adjusted for multiple comparisons followed all significant interactions. ^abc^ Significantly different at *p* < 0.05 using Tukey’s post hoc analysis. A *t*-test was used to compare HF and HF-MVM within each location. Significant differences (*p* < 0.05) are indicated by an asterisk (*). * *p* < 0.05, *** *p* < 0.001.

**Figure 2:**
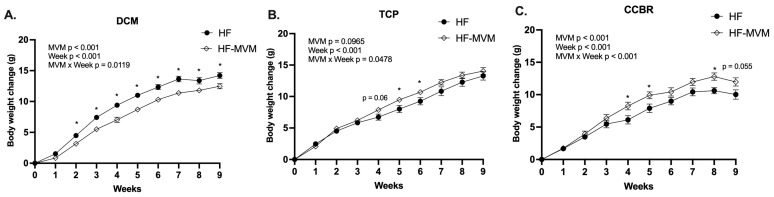
Body weight gain over time from week 1 to week 9 of the dietary intervention until termination in Location DCM (**A**), TCP (**B**), and CCBR (**C**). Values are Mean ± SEM, *n* = 9–12 for DCM and CCBR, and 12–15 in TCP. A two-way analysis of variance (ANOVA) was conducted by PROC MIXED procedure with MVM (non-MVM vs. MVM) and Week as main factors and an MVM × Week interaction term followed by Tukey’s post hoc test on body weight gain. Significant differences (*p* < 0.05) are indicated by an asterisk (*). * *p* < 0.05.

At the beginning of each experiment, there was no difference in the body weight either between treatments (MVM: *p* > 0.05 for all: DCM: HF 35.91 ± 1.01 g vs. MVM 35.92 ± 1.07 g; TCP: HF 34.53 ± 0.83 vs. MVM 34.72 ± 0.72; CCBR: HF 34.38 ± 1.05 vs. MVM 34.53 ± 1.05) or across the three locations (Location: *p* > 0.05) as well as their interaction (MVM*Location: *p* > 0.05). Body weight at week 9 and weight gain during the 9-week dietary intervention were affected by location as a main factor (*p* < 0.001, Figure 1A,B) and its interaction with the MVM (*p* < 0.05, Figure 1A). In the DCM, HF-MVM mice had lower weight gain and reduced BW compared to the control HF group (Figure 2A). However, in the TCP, HF-MVM mice had higher weight gain at weeks 4 (*p* = 0.06), week 5 (*p* < 0.05), and 6 (*p* < 0.05, Figure 2B). A similar pattern of increased weight gain over time was observed in the CCBR at weeks 4, 5, and 8 (*p* < 0.05), with a trend at week 9 (*p* = 0.055, Figure 2C). At week 9, mice from both DCM and TCP gained around 13 g, whereas mice from the CCBR had a lower gain of 11 g compared to the other two locations (Figure 1A, *p* < 0.05). At week 9, HF mice in DCM and HF-MVM mice in TCP were significantly heavier than HF mice in CCBR (*p* < 0.05, Figure 1B). Despite these differences, no significant variation in BW at termination was observed in either the TCP or CCBR (*p* > 0.05, Figure 1C).

### 3.2. Body Composition and Insulin Resistance

The eWAT mass, as well as its percentage of final body weight, were affected by the interaction between MVM and the location (*p* < 0.01 for both Figure 1D and Figure 1E). In the DCM, MVM increased the visceral adiposity index, primarily driven by increased eWAT weight and its percentage relative to body weight in HF diet-fed mice. However, in the TCP, despite the increased body weight gain over time compared to the HF group, HF-MVM mice had decreased eWAT when expressed as mass (*p* < 0.05) or relative to body weight percentage (*p* < 0.05). In the CCBR, the addition of MVM in the HF diet did not significantly affect these parameters (*p* > 0.05).

Liver mass and its percentage of final BW ratio were affected by the interaction between the effect of MVM and location (*p* < 0.01). These parameters were reduced in the HF-MVM group only in the DCM (*p* < 0.05, Figure 1F,G), while MVM had no effect in either the TCP or the CCBR on liver mass. (*p* > 0.05). HF-fed mice in TCP had smaller liver mass and liver/BW% than the HF group in DCM (*p* < 0.05).

**Figure 3:**
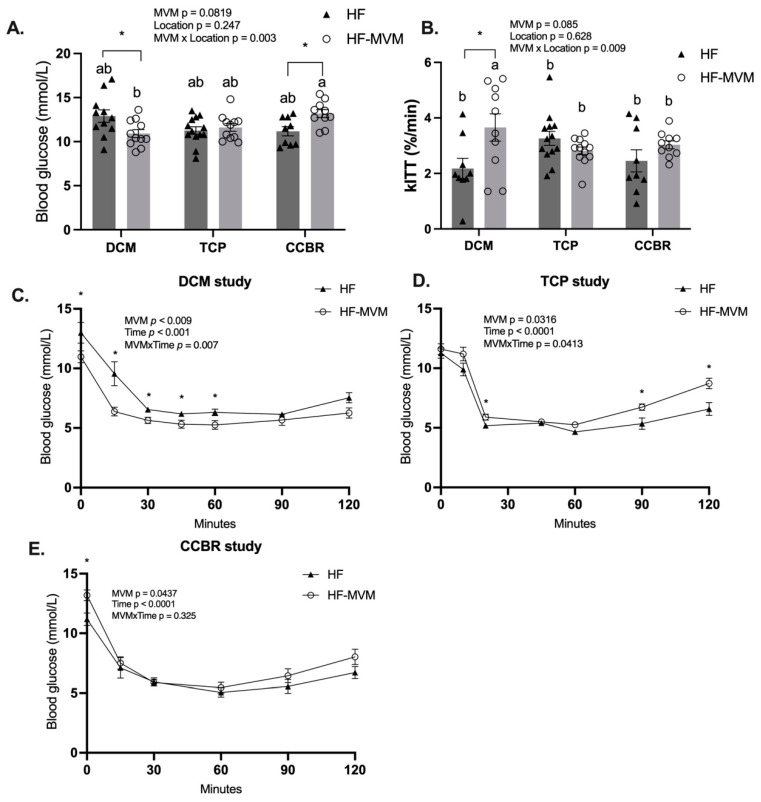
Insulin tolerance test. The fasting glucose level before insulin injection across three experiments (**A**); the glucose disappearance rate (kITT) from 0–15 min for DCM and CCBR, and 0–20 min after insulin injection across the three experiments (**B**); glucose levels after intraperitoneal injection of insulin from 0 to 120 min in DCM (**C**), TCP (**D**), and CCBR (**E**). Values are Mean ± SEM, *n* = 9–12 for DCM and CCBR, and *n* = 12–15 in TCP. A two-way analysis of variance (ANOVA) was conducted by PROC MIXED procedure with MVM (non-MVM vs. MVM) and time as main factors and an MVM*Time interaction term followed by Tukey’s post hoc test on ITT. A two-way ANOVA was conducted with dietary MVM and Location as main factors and an MVM × Location interaction term on fasting glucose and kITT by Tukey’s post hoc test. ^ab^ Significantly different at *p* < 0.05. * *p* < 0.05.

Fasting glucose was affected by the interaction between MVM and location across the three experiments (*p* < 0.01, Figure 3A). HF-MVM mice in DCM had the lowest fasting blood glucose, but HF-MVM mice in CCBR had the highest among the three experiments (*p* < 0.05). In each individual experiment, MVM decreased the fasting glucose level in mice in the DCM but increased it in the CCBR (*p* < 0.05, Figure 3A) compared to their HF controls.

Fasting insulin was affected by location (*p* < 0.01) and its interaction between location and MVM (*p* < 0.05, Figure 1H). HF mice in DCM had the highest insulin concentrations, which were nearly twice the mice in the other experiments (*p* < 0.05).

HOMA-IR, an indicator of insulin resistance, was affected by MVM (*p* < 0.05), location (*p* < 0.001), and their interaction (*p* < 0.001). In DCM, adding MVM reduced HOMA-IR by about 50% (*p* < 0.001); however, this effect was not observed in the other two locations (*p* > 0.05). Across the three experiments, mice in TCP were the least insulin resistant, while mice in HF mice in DCM had the highest HOMA-IR values (*p* < 0.01, Figure 1I).

### 3.3. Insulin Sensitivity

The MVM did not have a statistically significant effect on either fasting blood glucose (MVM effect: *p* = 0.0819, Figure 3A) or glucose disappearance rate (kITT) during the first 15 or 20 min after the injection (MVM effect: *p* = 0.085, Figure 3B). However, an interaction between MVM and location occurred (*p* < 0.01, Figure 3A,B). In all three experiments, HF-MVM in DCM had the lowest fasting glucose levels, while MVM-fed mice in CCBR had the highest level (*p* < 0.05, Figure 3A). Insulin sensitivity, indicated by the kITT values, was increased in HF-MVM mice in DCM when compared to mice in other locations (*p* < 0.05, Figure 3B).

The glucose concentrations over 120 min after insulin injection were affected by MVM as a main factor in each individual experiment (*p* < 0.05) and interacted (*p* < 0.05) with time in DCM and TCP (Figure 3C–E). In DCM, HF-MVM mice had decreased post-insulin-injection glucose compared to HF mice (Figure 3C), consistent with improved insulin sensitivity after MVM supplementation (*p* < 0.05, Figure 3B). In contrast, in the TCP, MVM-fed mice had increased glucose levels after insulin injection, primarily at 20 min, 90 min, and 120 min (*p* < 0.05). However, no interaction between MVM and time was observed in the CCBR, and MVM as a main factor decreased glucose response over time (*p* < 0.05, Figure 3E).

### 3.4. Gene Expression

**Figure 4:**
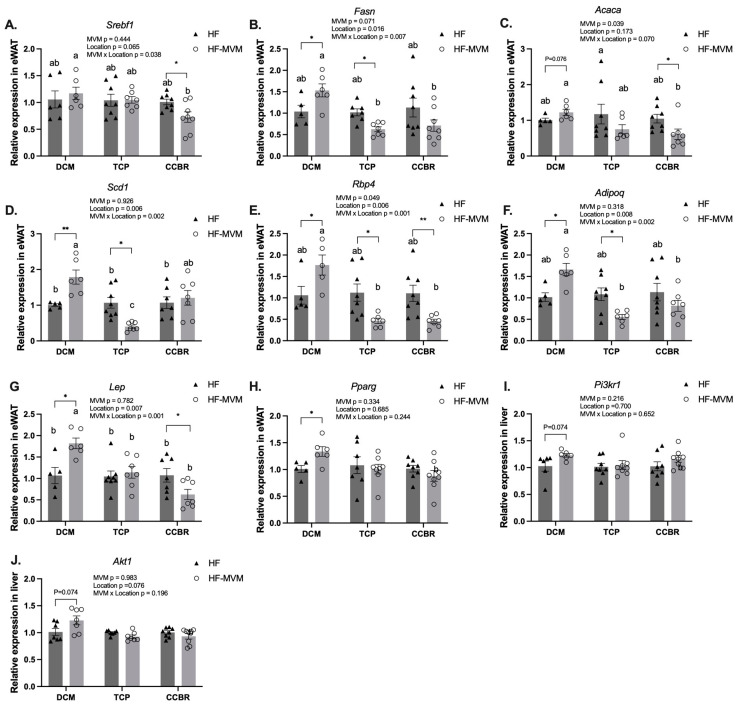
Relative mRNA expression of genes involved in lipogenesis (**A**–**D**), adipokine synthesis (**E**–**G**), and adipogenesis (**H**) in the eWAT, and insulin signaling (**I**,**J**) in the liver of mice in three experiments. Values are Mean ± SEM, *n* = 5–8/group. A two-way ANOVA was conducted with MVM (HF or HF-MVM) and Location (DCM, TCP, and CCBR) as the main factors and an MVM*Location interaction term. A Tukey’s post hoc analysis adjusted for multiple comparisons followed all significant interactions. ^abc^ Significantly different at *p* < 0.05 by Tukey’s post hoc analysis. A *t*-test was used to compare HF and HF-MVM within each location. Significant differences (*p* < 0.05) are indicated by an asterisk (*). * *p* < 0.05, ** *p* < 0.01.

In the three experiments, MVM affected the expression of three of the eight genes (*p* < 0.05, Figure 4) and showed an interaction between MVM and location for six of the eight genes involved in lipogenesis (Figure 4A–D), adipokine synthesis (Figure 4E–G), and adipogenesis (Figure 4H) pathways in the eWAT (*p* < 0.05, Figure 2). However, in the liver, neither a main effect from MVM nor its interaction with location was observed (Figure 4I,J).

In the lipogenesis pathway, *Srebf1*, *Fasn*, *Acaca*, and *Scd1* were measured. MVM affected the expression of *Acaca* (*p* < 0.05, Figure 4C), independent of location. *Srebf1*, *Fasn*, and *Scd1* were affected by location (*p* < 0.05) and interacted with MVM (*p* < 0.01, Figure 4A,B,D). Across the three experiments, HF-MVM mice in the DCM had the highest expression of *Srebf1*, whereas those in CCBR had the lowest (*p* < 0.05). For *Fasn*, HF-MVM mice in both TCP and CCBR had the lowest expression, but the DCM had the highest. HF-MVM mice in the DCM had the highest *Scd1* expression, four times higher than that in HF-MVM mice in TCP (*p* < 0.05, Figure 4D). In each experiment, *Fasn* expression was increased by MVM in DCM and decreased in TCP and CCBR. (*p* < 0.05). MVM upregulated the expression of *Scd1* over 70% in DCM (*p* < 0.05) but downregulated it by 40% in TCP (*p* < 0.05) and had no effect in CCBR. Both *Srebf1* and *Acaca* were downregulated by 30% and 40% in the eWAT of HF-MVM mice in CCBR (*p* < 0.05), but no effect from MVM in DCM or TCP was observed (*p* > 0.05, Figure 4A,C).

*Rbp4*, *Adipoq*, and *Lep*, three adipokine-encoding genes, were measured. MVM had a main effect on *Rbp4* (*p* < 0.05), and this effect was dependent on location (*p* < 0.01), indicated by an increase in the DCM but decreased expression in TCP and CCBR (Figure 4E). *Adipoq* and *Lep* were affected by location (*p* < 0.05) and its interaction with MVM (*p* < 0.01, Figure 4F,G). Across the three experiments, MVM-fed mice in DCM had the highest expression, and TCP and CCBR had the lowest expression of all three genes (*p* < 0.05). Within DCM, HF-MVM mice had about 60% upregulation in all three genes, compared to the HF group (*p* < 0.05 for all). In contrast, in both the TCP and CCBR, the inclusion of MVM to the HF diet downregulated the expression of *Rbp4* in eWAT by over 50% (*p* <0.05 and *p* < 0.01, Figure 4E). In HF-MVM mice, *Adipoq* was downregulated by more than 40% (*p* < 0.05, Figure 4F) in the TCP, and the expression of *Lep* was reduced by about 40% in the CCBR (*p* < 0.05), compared with HF mice (Figure 4G).

*Pparg*, a gene encoding an important adipogenesis factor, was not affected by MVM, location, or their interaction. The effect of MVM only existed in the DCM, where MVM increased the expression of the gene by 30% (*p* < 0.05, Figure 4H).

In the liver, *Pik3r1* and *Akt1* were selected based on their role in the insulin signaling pathway. Neither MVM, nor location, nor their interaction affected the expression of these two genes (*p* > 0.05, Figure 4I,J).

## 4. Discussion

The results of the study support our hypothesis that MVM would have physiological effects in all experimental locations. In our previous study, adding MVM to an HF diet fed to adult mice reduced weight gain and IR, modified the expression of genes associated with IR in white adipose tissue, and shifted hepatic one-carbon metabolism toward favoring increased gene methylation [16]. In the present study, we show that the direction and magnitude of MVM effects were modified by the environment of the experimental location and its associated factors. These results support the call for the reproducibility of results in different experimental settings before conclusions are drawn and applied from preclinical research [1,2,3].

To our knowledge, this is the first report to demonstrate that the effects of micronutrients on physiological phenotypes and gene expression were modified by the heterogeneity of the environment. Our results are consistent with other reports that environmental variations between animal facilities affect experimental outcomes despite attempts to minimize the differences [4].

In all three experiments, although we maintained the genetic backgrounds of the mice and employed similar experimental study designs, the effects of MVM on BW, fat mass, insulin sensitivity, and resistance were dependent on the experimental location. MVM additions decreased BW gain in one (DCM) but increased it in the other two locations (TCP and CCBR). In DCM, the reduced BW and weight gain by MVM were associated with an increase in eWAT mass, improved insulin sensitivity, and lower IR. Conversely, in the TCP and CCBR, mice fed with the HF-MVM diet had decreased responses in these outcomes. In DCM, the results of ITT and HOMA-IR confirmed lower IR and were consistent with lower body weight. Conversely, in TCP and CCBR, these measures showed higher IR in those with higher body weight in response to MVM. These observations demonstrate a strong effect of MVM on obese mice; however, it was also determined by the experimental environment.

The effects of MVM on fat mass and the expression of six genes expressed in eWAT also interacted with the experimental location. Reduced IR in DCM was associated with higher expression of *Fasn*, *Rbp4*, *Scd*, *Adipoq*, and *Lep* in eWAT when compared to HF mice. However, in the TCP location, mice fed the HF-MVM diet had decreased eWAT mass and percentage, and these changes were associated with lower expression of these genes. Similarly, in the CCBR, although eWAT mass did not differ, the expression of *Srebf1*, *Acaca*, *Rbp4*, and *Lep* was lower. Together, these results indicate that the effect of MVM was dependent not only on the experimental location at the level of phenotype but also on gene expression in the eWAT. This location-dependent effect of MVM on gene expression indicates that the alteration is likely to be associated with epigenetic changes induced by heterogeneous experimental environments.

From all genes investigated, MVM had a main and consistent effect on two genes related to adipose function in eWAT in all locations, but it also interacted with the location, which was also a main factor. *Rbp4*, responsible for encoding retinol-binding protein, was similarly affected by MVM, and it was dependent on location. *Fasn*, which encodes fatty acid synthase, was upregulated in DCM and downregulated in TCP and CCBR.

Retinol-Binding-Protein 4 (*Rbp4*) not only serves as a transport protein for retinol but also exerts an autocrine/paracrine function in adipose tissue. The progression of obesity often leads to elevated secretion of *Rbp4* by adipocytes, impairing insulin sensitivity and accelerating lipolysis in adipose tissue [20]. In the DCM, a 2.2-fold higher expression of *Rbp4* was found in the eWAT of HF-MVM mice compared to HF mice, which corresponded to the enlargement of their eWAT. However, in both the TCP and CCBR, HF-MVM mice had lower expression of this gene, and this was associated with their reduced eWAT weight and percentage. Notably, the effect of MVM on *Rbp4* expression is consistent across the three experiments, and it was dependent upon the proportion of eWAT/BW%. Therefore, the effect of MVM on the expression of *Rbp4* may be mediated by body weight and/or body composition, which are sensitive to food intake [21].

Fatty acid synthase (*Fasn*) is needed for the production of long-chain fatty acids from acetyl-CoA and malonyl-CoA, representing one of the rate-limiting steps in de novo lipogenesis [22]. In the DCM, *Fasn* expression in eWAT was not only associated with eWAT weight but also showed an inverse association with body weight and a trend for body weight gain in HF-MVM mice. These association patterns remained consistent across experiments. Furthermore, irrespective of the direction of gene regulation and the disparities in phenotypic outcomes among the three experiments, the addition of MVM to an HF diet consistently affected gene expression in eWAT. These results provide evidence that micronutrient intake is a factor modulating gene expression in the white adipose tissue in adulthood. Beyond micronutrients, other food components can also affect the expression of the gene. For instance, Chaplin et al. reported that the expression of *Fasn* in eWAT was upregulated in mice fed an HF diet supplemented with conjugated linoleic acid (CLA) and CLA + calcium, but not calcium alone, compared to the HF control group [23]. Furthermore, resveratrol, a polyphenol found in grape skin, restored *Fasn* gene expression from the perturbed levels induced by an HF diet. This restoration was accompanied by DNA methylation status for the gene [24]. Altogether, these results may suggest responsiveness or plasticity of *Fasn* gene expression in response to nutrients in the diet.

It is clear that the MVM addition, while location-dependent, affected both obese phenotype and gene expression in heterogeneous experimental environments. Many factors may have modified the effect of the MVM on the characteristics of the metabolic syndrome, as summarized in the Supplemental Table S5. Although all mice were from the same breeding company/site, variations in the maternal diets may have influenced the epigenetic baselines of the experimental animals [25]. The different number of mice housed per cage could have created different hierarchical structures among male mice, with variations in circulating testosterone levels between dominant and subordinate males influencing their behaviors and metabolism [26]. The sex of experimenters is also known to impact the stress levels of the mice [13,27], which may have modified mice’s behavior and metabolism. Other environmental sources of biological variations, including holding room occupancy, cage location, and overall day-to-day activity in the test location on-site, may have impacted the animal epigenome and physiological phenotypes [4].

The lack of individual animal food intake was a major limitation of the study, thus limiting the interpretation of the origin of the physiological outcomes. Individual food intake was not measured due to our Research Ethics Boards’ mandatory group housing regulation. Although the content of micronutrients added to the diets was confirmed and showed consistency in the three locations and across the diet batches, other aspects, including diet storage, may have played a role. SFAs, MUFAs, and, particularly, N-6 PUFAs differed among the different experimental locations as well as within batches of diet from the same experiment (HF vs. HF-MVM) (Supplemental Table S3). PUFAs are susceptible to degradation. Lard, a major component of HF diets, contains a significant proportion of unsaturated fatty acids, which are prone to oxidization [28]. Lipid peroxidation in the TCP diets was two to three times higher than in the DCM and CCBR diets (Supplemental Table S2). Because mice possess exceptional sensory abilities in olfactory and gustatory perceptions [29], even slight modifications in these sensory cues can significantly affect their food intake and possibly induce stress responses [30].

Although the primary environmental factors modifying study outcomes have not been identified, these results provide evidence that MVM additions to the high-fat diet influence obesity and characteristics of the metabolic syndrome of adult mice in three different environments. The observation is of significance to the design of dietary interventions in obese adults with characteristics of metabolic syndrome. Blood measures of micronutrients, including vitamins A, B1, B6, and B12, as well as the trace minerals Se and Zn, have been reported to be suboptimal in these individuals [31,32,33]. Many of these have been added to the diets as supplements in randomized controlled trials and provided the background for the content of the MVM [34,35]. However, the beneficial results of micronutrient supplementation for improving metabolic health in obese adults appear to be inconsistent [36]. Our findings provide a possible explanation for the variability in outcomes of the human trials conducted to date [36], suggesting that differences in the living conditions of selected participants need attention. Also, by identifying potential mechanisms, the results add plausibility to the fact that micronutrient supplements modify the metabolic consequences of obesity and thus may encourage the design of such studies.

## 5. Conclusions

MVM supplementation influenced phenotypes and expression of genes related to adipose function in obese adult male mice, but the experimental location and its associated conditions were significant interacting factors. Preclinical studies investigating the relationship between diet and metabolic outcomes should acknowledge the plasticity of the epigenome and implement measures to reproduce studies in different locations. If clinical studies are to be derived from preclinical outcomes, greater efforts should be made to mitigate the impact of environmental variables on participants in clinical studies in free-living conditions.

## Data Availability

Data is available from the research team upon reasonable request.

## Supplementary Materials

The following supporting information can be downloaded at: https://www.mdpi.com/article/10.3390/nu16050696/s1, Table S1: Composition of diets used in the three experiments; Table S2: Expected and measured micronutrient concentrations and peroxidation values in the experimental diets; Table S3: Free fatty acids analyses of the three experimental diets; Table S4: TaqMan^®^ Gene Expression Assays; Table S5: Potential variables (experimental factors) among three experiments.
