# Supplementary material for: The Effect of Micronutrients on Obese Phenotype of Adult Mice Is Dependent on the Experimental Environment
Source: Nutrients. 2024 Feb 29;16(5):696. doi: 10.3390/nu16050696 (PMC10935069; doi:10.3390/nu16050696)
Supplement: Supplementary file 1 [file nutrients-16-00696-s001.zip › nutrients-2847477-supplementary.pdf]

Supplemental Table 1: Composition of diets[1] used in the three experiments

	HF	HF-MVM
	gm% (kcal%)	
Protein	**26.2** (20)	**26.2** (20)
Carbohydrate	26.3 (20)	25 (20)
Fat	**34.9** (60)	**34.9** (60)
Ingredient	*gm (kcal)*	
Casein	**200** (800)	**200** (800)
L-Cystine	**3** (12)	**3** (12)
Maltodextrin 10	**125** (500)	**125** (500)
Sucrose	**68.8** (275)	**68.8** (275)
Cellulose, BW200	**50** (0)	**50** (0)
Soybean Oil	**25** (225)	**25** (225)
Lard	**245** (2205)	**245** (2205)
Dicalcium Phosphate	**13** (0)	**13** (0)
Calcium Carbonate	**5.5** (0)	**5.5** (0)
Potassium Citrate, 1 H_2O	**16.5** (0)	**16.5** (0)
Mineral Mix (S10026)[2]	**10** (0)	-
Selenium (mg)	**0.16 (0)**	-
Zinc (mg)	**29** (0)	-
Mineral Mix (S10110)[3]	-	**10** (0)
Selenium (mg)	-	**0.32** (0)
Zinc (mg)	-	**145** (0)
Vitamin Mix (V10001)[4]	**10** (40)	**0**
Vitamin A (IU)	**4000** (0)	-
Vitamin B_1 (mg)	**6.0** (0)	-
Vitamin B_6 (mg)	**7.0** (0)	-
Vitamin B_{12} (mg)	**0.010** (0)	-
Vitamin Mix (V10103)[5]	-	**10** (40)
Vitamin A (IU)	-	**20000** (0)
Vitamin B_1 (mg)	-	**30.0** (0)
Vitamin B_6 (mg)	-	**35.0** (0)
Vitamin B_{12} (mg)	-	**0.050** (0)
Choline Bitartrate (g/kg)	**2** (0)	**2** (0)
Total	**773.85** (4057)	**773.85** (4057)

1. All experimental diets were purchased from Research Diets in New Brunswick, NJ, USA. Micronutrient forms were as follows: Vitamin A: retinol acetate; Vitamin B1: thiamin HCl (78.8% thiamin); Vitamin B6: pyridoxine-HCl (82% pyridoxine); Vitamin B12: cyanocobalamin, 0.1%; Zn: Zinc Carbonate (52.1% Zn); Se: Sodium Selenite (45.7% Se).
2. The Mineral Mix (S10026, https://researchdiets.com/formulas/S10026B).

3. The Mineral Mix (S10110) was a customized mix with a total of 5-fold Zn and 2-fold Se, while other minerals remained consistent with the Mineral Mix (S10026).
4. The Vitamin Mix (V10001, https://researchdiets.com/formulas/V10001).
5. The Vitamin Mix (V10103) was a customized mix with a total of 5-fold vitamin A, B1, B6, and B12, while other vitamins remained consistent with those in the Vitamin Mix (V10001).

Confirmation of Diet Composition

1. Total micronutrient concentrations

The analysis of vitamin A, B1, B6, B12, Zn, and Se in the HF diet, as well as vitamin A, B1, B6, B12, and Zn in the HF-MVM diet, closely approximated the expected values (Supplemental Table 2). The micronutrients were not affected by radiation exposure in the TCP. However, the measured values of Se in the HF-MVM diet were not consistent among the three experiments, perhaps reflecting its unequal distribution during mixing of the diets.

2. Lipid peroxidation values

The HF diet (Cat#D12492, Research Diet) contained lard, which is rich in saturated (37%) and monounsaturated (~46%) as well as polyunsaturated (~17%) fatty acids and susceptible to oxidation (Rendina-Ruedy E, Smith BJ. Methodological considerations when studying the skeletal response to glucose intolerance using the diet-induced obesity model. *Bonekey Rep*. 2016; 5:845) and is susceptible to rancidity potentially affecting palatability and food intake of the mice. In TCP, both the HF and HF-MVM diets exposed to radiation exhibited peroxidation values approximately three times higher than those observed in the DCM diets (TCP: 6.3 and 6.2 meq/kg vs. 2.1 and 2.0 meq/kg, Supplemental Table 2). Additionally, the values were approximately twice as high as those recorded in the CCBR diets (TCP: 6.3 and 6.2 meq/kg vs. CCBR: 3.0 and 2.9 meq/kg).

Supplemental Table 2. Expected and measured micronutrient concentrations and peroxidation values in the experimental diets

**Nutrient to analyze**	**Expected values**		**Measured values**					
			DCM		TCP		CCBR	
	HF	MVM	HF	MVM	HF	MVM	HF	MVM
Vitamin A (IU/100g)	400	2000	-	-	534	1930	414	1440
Vitamin B1 (mg/100g)	0.75	3.75	-	2.96	0.454	2.69	-	-
Vitamin B6 (mg/100g)	0.88	4.38	-	3.58	0.589	3.51	-	-
Vitamin B12 (ug/100g)	1.25	6.25	-	6.01	0.87	5.15	-	-
Zn (ppm)	36.25	181.25	-	201	40	211	-	-
Se (ppm)	0.2	0.4	-	0.736	0.235	0.786	0.226	0.516
Peroxidation value (meq/kg)			2.1	2.0	6.3	6.2	3	2.9

Free fatty acids analyses of the three experimental diets

Free fatty acid (FFA) composition, including saturated fatty acids (SFAs), monounsaturated fatty acids (MUFAs), and omega-3 (N-3) and omega-6 (N-6) polyunsaturated fatty acids (PUFAs), were determined. (Supplemental Table 4). No differences were detected in the concentration of total fatty acids or in the ratio between N-6 and N-3 PUFAs due to treatment or location ($P > 0.05$, Supplemental Table 4). However, N-6 PUFAs were significantly affected by MVM, location, and their interaction ($P < 0.05$). In both TCP and CCBR, HF diets had higher levels of N-6 PUFA than HF-MVM diets ($P < 0.05$, Supplemental Table 4), but not in DCM. These results were driven primarily by differences in the effects of HF and HF-MVM diets ($P < 0.05$) and by location ($P < 0.05$) and their interactions ($P < 0.05$) on the PUFAs C 20:3n-6, C 20:4n-6, and C 22:2n-6. C 20:3n-3, one of the N-3 PUFAs, was affected by treatment ($P < 0.01$) and its interaction with location ($P < 0.05$). The HF diet in DCM and the HF-MVM diet in TCP and CCBR had the lowest levels of this N-3 PUFA, but the HF diet in CCBR had more than twice the amount than them ($P < 0.05$, Supplemental Table 4).

Additionally, within the SFAs category, C 20:0 was affected by treatments ($P < 0.05$) and location ($P < 0.05$). Both HF-MVM diets in TCP and CCBR had 40% and 30% lower C 20:0 than their control HF diets, respectively ($P < 0.05$ for both).

In the MUFAs, only C 18:1n-7 was affected by treatment, location, and their interaction ($P < 0.05$). It was ten-fold lower in the HF-MVM diet in the TCP than in any other locations ($P < 0.05$).

Supplemental Table 3. Free fatty acids analyses of the three experimental diets

ug/mL	Treatment	Location			P values		
		DCM	TCP	CCBR	**MVM**	**Location**	**MVM *Location**
**SFAs**	HF	125.82±26.92	125.87±13.92	103.56±6.43	0.286	0.300	0.940
	HF-MVM	137.1±22.76	148.7±7.69	116.99±18.53			
C12:0	HF	0.19±0.04	0.17±0.07	0.15±0.06	**0.080**	0.914	0.942
	HF-MVM	0.28±0.02	0.25±0.02	0.27±0.12			
C14:0	HF	2.76±0.71	2.68±0.54	2.13±0.31	0.247	0.380	0.665
	HF-MVM	2.28±0.72	3.78±0.19	2.69±0.87			
C16:0	HF	63.21±14.61	63.36±8.42	48.16±2.56	0.186	0.171	0.716
	HF-MVM	66.65±3.82	83.02±3.81	58.7±12.5			
C18:0	HF	58.32±11.33	57.84±9.18	50.72±5.94	0.462	0.397	0.932
	HF-MVM	65.91±16.88	60.61±6.51	53.74±10.67			

C20:0	HF	1.33±0.33	1.81±0.32	2.43±0.11	**0.012**	**0.020**	0.080
	HF-MVM	1.44±0.12	0.95*±0.2	1.61*±0.16			
**MUFAs**	HF	169.04±34.22	202.1±21.16	159.42±4.08	0.352	0.149	0.990
	HF-MVM	188.59±27.38	221.33±13.19	173.25±22.92			
C18:1n-7	HF	10.99^{a}±1.77	13.15^{a}±1.08	9.52^{a}±1.36	**0.028**	**0.003**	**0.001**
	HF-MVM	13.2^{a}±0.83	0.95^{b}±0.2	10.97^{a}±1.17			
C18:1n-9	HF	154.85±31.13	188.02±23.95	147.85±5.88	0.263	**0.090**	0.910
	HF-MVM	175.86±22.54	217±13.08	158.55±20.50			
C20:1n-9	HF	3.2±0.85	5.01±0.68	4.93±0.38	0.115	0.281	0.178
	HF-MVM	3.7±0.39	3.36±0.54	3.73±0.37			
**N-6s**	HF	214.27^{bc}±24.72	262.9^{ab}±30.0	342.46^{a}±41.19	**0.006**	**0.017**	**0.005**
	HF-MVM	263.19^{ab}±6.22	141.59^{c*}±6.89	222.05^{bc*}±11.78			
C18:2n-6	HF	85.36±17.43	102.37±9.72	79.67±3.75	0.261	0.157	0.987
	HF-MVM	98.37±14.50	113.43±7.12	89.0 ±11.38			
C20:2n-6	HF	3.28±1.27	3.52±0.40	3.99±0.52	0.651	0.639	0.898
	HF-MVM	3.82±1.25	3.37±0.38	4.86±0.52			
C20:3n-6	HF	0.73^{ab} ±0.24	1.18^{ab}±0.16	1.4^{a}±0.15	**0.016**	0.821	**0.040**
	HF-MVM	0.93^{ab}±0.35	0.5^{b*}±0.07	0.49^{b*}±0.13			
C20:4n-6	HF	1.45±0.40	2.13±0.25	2.32±0.12	**0.020**	0.600	**0.092**
	HF-MVM	1.62±0.31	1.11*±0.17	1.32*±0.34			
C22:2n-6	HF	118.36^{b} ±28.9	144.53^{ab}±27.75	231.48^{a}±28.8	**0.003**	**0.004**	**0.007**
	HF-MVM	150.57^{ab}±15.03	22.2^{c*}±5.95	121.52^{b}±14.46			
C22:4n-6	HF	5.10±4.65	9.17±6.24	23.6±7.13	**0.066**	0.171	0.129
	HF-MVM	7.87±5.47	0.97±0.23	4.87±1.16			
**N-3s**	HF	12.01±3.06	17.9±6.44	27.09±8.02	**0.090**	0.614	0.110
	HF-MVM	15.75±4.34	10.89±0.54	9.27±1.07			
C18:3n-3	HF	7.72±1.32	9.01±0.43	7.76±0.59	0.210	0.248	0.776
	HF-MVM	9.37±1.40	10.1±0.47	8.06±0.91			

C20:3n-3	HF	0.98[b] ±0.27	1.6[ab] ±0.37	2.13[a]±0.07	**0.003**	**0.085**	**0.033**
	HF-MVM	1.09[ab]±0.07	0.58[b]±0.07	0.97[b*]±0.15			
**N-6/N-3**	HF	19.40±3.33	17.00±3.30	15.08±4.60	0.594	0.320	0.151
	HF-MVM	18.80±3.91	12.82±0.34	24.28±2.53			
**Total**	HF	521.14±62.77	608.77±41.54	632.53±56.24	0.329	0.948	0.109
	HF-MVM	604.63±40.69	522.51±25.09	521.56±43.29			

Values are Mean ± SEM, n = 3/group (analyzing in triplication). A two-way ANOVA was conducted with MVM (HF or HF-MVM) and Location (DCM, TCP, and CCBR) as main factors and an MVM*Location interaction term. A Tukey's post-hoc analysis adjusted for multiple comparisons followed all significant effects. [abc]Significantly different at P<0.05 by Tukey's post-hoc analysis. A t-test was used to compare the difference between HF and HF-MVM groups within each Location. Significant differences (P<0.05) are indicated by an asterisk (*). *P < 0.05.

Supplemental Table 4 TaqMan® Gene Expression Assays

Gene	Assay ID
**Adipogenesis**	
Peroxisome proliferator-activated receptor gamma, *Pparg*	Mm00440940_m1
**Adipokine Synthesis**	
Leptin, *Lep*	Mm00434759_m1
Adiponectin, *Adipoq*	Mm00456425_m1
Retinol binding protein 4, *Rbp4*	Mm00803266_m1
**Lipid Metabolism**	
Sterol regulatory element binding factor 1, *Srebf1*	Mm00550338_m1
Fatty acid synthase, *Fasn*	Mm00662319_m1
Stearoyl-CoA Desaturase, *Scd*	Mm00772290_m1
Acetyl-CoA Carboxylase alpha, *Acaca*	Mm01304257_m1
**Insulin signaling Pathway**	
Phosphoinositide-3-kinase regulatory subunit 1, *Pik3r1*	Mm01282781_m1
AKT serine/threonine kinase 1, *Akt1*	Mm01331626_m1
**Housekeeping gene**	
TATA box binding protein, *Tbp*	Mm00446973_m1

Supplemental Table 5. Potential variables (experimental factors) among three experiments.

	DCM	TCP	CCBR
**Maternal diet**	?	?	?
**Dietary raw ingredient**	?	?	?
**Dietary peroxidation levels**	2 meq/kg	6.3 meq/kg	3 meq/kg
**Dietary free fatty acids**	Notable differences in specific SFAs, MUFAs, and PUFAs among diets from different Locations (DCM vs. TCP vs. CCBR) and even within the same Location (HF vs. HF-MVM)		
**Experimenters' sex**	Only males	Males and females	Males and females
**Facility staff's sex**	Female	Male	Female
**Experimental period**	2020 Dec- 2021 March	2022 Feb-May	2022 July-October
**Mice per cage**	4	5	4
**Occupancy of the room**	Quiet, not busy	Busy	Busy and crowded
**Location of the cages in the holding rack**	Middle of the rack	Middle of the rack	Close to the bottom
**Location of the rack**	Facing the wall	Facing another rack	Facing the operating station
**Location of the facilities**	Considering the distance from subway trains and/or constriction sites		
